# 3D bioprinting of cells, tissues and organs

**DOI:** 10.1038/s41598-020-70086-y

**Published:** 2020-08-18

**Authors:** Madhuri Dey, Ibrahim T. Ozbolat

**Affiliations:** 1 Department of Chemistry, Penn State University, University Park, PA 16802 USA; 2 The Huck Institutes of the Life Sciences, Penn State University, University Park, PA 16802 USA; 3 Engineering Science and Mechanics Department, Penn State University, University Park, PA 16802 USA; 4 Biomedical Engineering Department, Penn State University, University Park, PA 16802 USA; 5 Materials Research Institute, Penn State University, University Park, PA 16802 USA

**Keywords:** Tissue engineering, Experimental models of disease, Regeneration

## Abstract

3D bioprinting has emerged as a promising new approach for fabricating complex biological constructs in the field of tissue engineering and regenerative medicine. It aims to alleviate the hurdles of conventional tissue engineering methods by precise and controlled layer-by-layer assembly of biomaterials in a desired 3D pattern. The 3D bioprinting of cells, tissues, and organs Collection at *Scientific Reports* brings together a myriad of studies portraying the capabilities of different bioprinting modalities. This Collection amalgamates research aimed at 3D bioprinting organs for fulfilling demands of organ shortage, cell patterning for better tissue fabrication, and building better disease models.

The discovery of a 3D printer dates back to early 1980s when Charles Hull, an American engineer, built the 1st 3D printer, capable of creating solid objects by following a computer-aided design (CAD). The printer deposited successive layers of an acrylic-based photopolymer which was then simultaneously crosslinked by UV light, thus creating a solid 3D object. This simple technology, called stereolithography (SLA), revolutionized the additive manufacturing industry. Gradually, by the late 1990s, 3D printing made its appearance in healthcare where surgeons began 3D printing dental implants, custom prosthetics, and kidney bladders. Subsequently the term ‘3D bioprinting’ emerged where the material being printed, called ‘bioink’^1^^,^ consisted of living cells, biomaterials, or active biomolecules. Analogous to additive manufacturing, 3D bioprinting involves layer-by-layer deposition of bioink to create 3D structures, such as tissues and organs^2^.

3D bioprinting can be broadly categorized as either extrusion^3^^,^ droplet^4^^,^ or laser-based bioprinting. Extrusion based bioprinting employs mechanical, pneumatic or solenoid dispenser systems to deposit bioinks in a continuous form of filaments, while droplet based bioprinting relies on the generation of bioink droplets by thermal, acoustic or electrical stimulation. Laser based bioprinting utilizes laser power to 3D print structures such as in SLA by a photopolymerization principle. It can also be used for precise positioning of cells such as in laser direct-write and Laser Induced Forward Transfer (LIFT). The selection of “bioinks” for each of these different bioprinting modalities usually varies based on the ink’s rheology, viscosity, crosslinking chemistry, and biocompatibility. Extrusion based bioprinting primarily requires shear thinning bioinks while droplet or inkjet bioprinting needs materials with low viscosity. Over the past few years, the design and synthesis of bioinks has evolved to meet the increasing needs of new bioprintable materials. Significant advancements have also been made to integrate secondary techniques accompanying the above-mentioned modalities of bioprinting. For example, creating 3D structures with low viscosity bioinks has always been a challenge. To overcome this issue, such bioinks can now be extruded in a granular support bath containing yield stress hydrogels which solidify around the extruded structure and prevent it from collapsing^5^. Apart from organ printing, bioprinting is also being used to fabricate in-vitro tissue models for drug screening, disease modelling, and several other in-vitro applications.

The 3D bioprinting of cells, tissues and organs Collection at *Scientific Reports* is dedicated to this field of research. This collection clearly portrays the diverse applications of different bioprinting modalities and how they could be utilized for improving various aspects of healthcare. Kim et al. 3D printed a novel two-layered polycaprolactone (PCL) -based tubular tracheal graft^6^. This tracheal graft, seeded with induced pluripotent stem cell (iPSC) -derived mesenchymal (MSCs) and chondrocyte stem cells supported the regeneration of tracheal mucosa and cartilage in a rabbit model of a segmental tracheal defect. Galarraga et al. used a norbornene-modified hyaluronic acid (NorHA) macromer as a representative bioink for cartilage tissue engineering^7^. Printed structures containing MSCs, on long term culture, not only led to an increase in compressive moduli, but also expressed biochemical content similar to native cartilage tissue. Vidal et al. used 3D printed customized calcium phosphate scaffolds with and without a vascular pedicle to treat large bone defects in sheep^8^. They used CT angioscan to scan the entire defect site and subsequently 3D print a personalized scaffold to anatomically fit the defect site. A bioink comprising decellularized matrix from mucosal and muscular layers of native esophageal tissues was used by Nam et al. to mimic the microenvironment of native esophagus^9^. Leucht et al. used gelatin based bioinks to study vasculogenesis in a bone-like microenvironment^10^. Kilian et al. used a calcium phosphate cement (CPC) and an alginate-methylcellulose based bioink containing primary chondrocytes to mimic the different layers of osteochondral tissue^11^.

This special issue also contains three notable research articles on the patterning of cells—two utilizing acoustics, and one, magnetism. Even though bioprinting enables the homogenous distribution of cells representing the macro-architectural properties, it lacks control of the tissue micro-architecture such as orientation of cells within the bioprinted constructs. Chansoria and Shirwaiker delved deep into the physics of ultrasound-assisted bioprinting (UAB) that utilizes the acoustophoresis principle to align MG63 cells within single and multi-layered extrusion-bioprinted alginate constructs^12^. Cells were aligned both orthogonally and in parallel to the printed filaments, thus mimicking cellular anisotropy in tissues such as ligaments, tendons, and cardiac muscle. Similarly, Sriphutkiat et al. used acoustic excitation to align skeletal myoblast cells (C2C12) and human umbilical vein endothelial cells (HUVECs) encapsulated in methacrylated gelatin (GelMA) bioink^13^. Goranov et al. magnetically labelled MSCs and HUVECs, and aligned them in a magnetic scaffold to mimic vascularization of bone constructs^14^.

It is important to note that the applications of 3D bioprinting are not limited to organ printing. It also holds great promise in less explored avenues, such as using scaffolds for drug delivery, studying disease mechanisms, or creating personalized medicines. In this Collection, Lee et al. 3D printed a rifampicin loaded PCL scaffold for possible treatment of osteomyelitis^15^. Xu and coworkers 3D printed paracetamol containing PVA tablets with three different geometries, each demonstrating different release profiles which could be tailored based on the patient's needs^16^. Further, Foresti et al. applied 5D additive manufacturing techniques to create personalized models of patients’ pathology^17^. Ding, Illsley and Chang 3D bioprinted GelMA-based models to investigate the trophoblast cell invasion phenomenon, enabling studies of key placental functions^18^.

Additionally, there are other notable articles in this Collection enumerating different aspects of bioprinting. Afghah et al. used a Pluronic-nanoclay based composite support bath to bioprint representative structures, for complex and hollow tissues, using cell laden alginate hydrogel^19^. Zhao et al. developed a 3D printed hanging drop dripper system for analyzing tumor spheroids in-situ^20^. Yumoto et al. performed RNA-seq analysis on inkjet-printed cells to analyze the effect of bioprinting on gene expression^21^. We would like to extend our utmost gratitude and thank all the authors and reviewers who devoted their time and effort towards this 3D bioprinting collection.

Even though 3D bioprinting is advancing at a commendable rate with researchers trying to develop new printing modalities as well as improve existing modalities, there still remains a multitude of challenges that need to be overcome. Currently, a limited number of bioinks exist which are both bioprintable and which accurately represent the tissue architecture needed to restore organ function post-printing. While bioinks made from naturally derived hydrogels are conducive to cell growth, synthetic hydrogels are mechanically robust. Thus, hybrid bioinks should be designed to amalgamate all these aspects. Moreover, the bioprinting process itself needs to be more cell-friendly. Shear stress applied to the cells during the printing process are detrimental to cell growth and might even alter the gene expression profiles. Stem cells, such as iPSCs, are sensitive to such physical forces and usually do not survive the printing process. As stem cell studies have mostly been performed on 2D environments, there exists a lot of unknowns for a 3D stem cell culture. Effective techniques need to be developed for high throughput generation and bioprinting of organoids^22^ for personalized drug testing and predictive disease models. Additionally, vascularization of bioprinted constructs for proper nutrient exchange, as well as integration of printed vasculature with host vasculature post organ implantation, is another major obstacle. Overall, 3D bioprinting is a rapidly evolving field of research with immense challenges, but tremendous potential to revolutionize modern medicine and healthcare.

## References

[1] Hospodiuk M, Dey M, Sosnoski D, Ozbolat IT (2017). The bioink: a comprehensive review on bioprintable materials. Biotechnol. Adv..

[2] Ozbolat IT (2016). 3D Bioprinting: Fundamentals, Principles and Applications.

[3] Ozbolat IT, Hospodiuk M (2016). Current advances and future perspectives in extrusion-based bioprinting. Biomaterials.

[4] Gudapati H, Dey M, Ozbolat I (2016). A comprehensive review on droplet-based bioprinting: past, present and future. Biomaterials.

[5] Heo DN (2020). 3D bioprinting of carbohydrazide-modified gelatin into microparticle-suspended oxidized alginate for the fabrication of complex-shaped tissue constructs. ACS Appl. Mater. Interfaces.

[6] Kim IG (2020). Transplantation of a 3D-printed tracheal graft combined with iPS cell-derived MSCs and chondrocytes. Sci. Rep..

[7] Galarraga JH, Kwon MY, Burdick JA (2019). 3D bioprinting via an in situ crosslinking technique towards engineering cartilage tissue. Sci. Rep..

[8] Vidal L (2020). Regeneration of segmental defects in metatarsus of sheep with vascularized and customized 3D-printed calcium phosphate scaffolds. Sci. Rep..

[9] Nam H (2020). Multi-layered free-form 3D cell-printed tubular construct with decellularized inner and outer esophageal tissue-derived bioinks. Sci. Rep..

[10] Leucht A, Volz AC, Rogal J, Borchers K, Kluger PJ (2020). Advanced gelatin-based vascularization bioinks for extrusion-based bioprinting of vascularized bone equivalents. Sci. Rep..

[11] Kilian D (2020). 3D Bioprinting of osteochondral tissue substitutes-in vitro-chondrogenesis in multi-layered mineralized constructs. Sci. Rep..

[12] Chansoria P, Shirwaiker R (2019). Characterizing the process physics of ultrasound-assisted bioprinting. Sci. Rep.

[13] Sriphutkiat Y, Kasetsirikul S, Ketpun D, Zhou Y (2019). Cell alignment and accumulation using acoustic nozzle for bioprinting. Sci. Rep..

[14] Goranov V (2020). 3D patterning of cells+in magnetic scaffolds for tissue engineering. Sci. Rep..

[15] Lee JH (2020). Development of a heat labile antibiotic eluting 3D printed scaffold for the treatment of osteomyelitis. Sci. Rep..

[16] Xu X, Zhao J, Wang M, Wang L, Yang J (2019). 3D printed polyvinyl alcohol tablets with multiple release profiles. Sci. Rep..

[17] Foresti R (2020). In-vivo vascular application via ultra-fast bioprinting for future 5D personalised nanomedicine. Sci. Rep..

[18] Ding H, Illsley NP, Chang RC (2019). 3D bioprinted GelMA based models for the study of trophoblast cell invasion. Sci. Rep..

[19] Afghah F, Altunbek M, Dikyol C, Koc B (2020). Preparation and characterization of nanoclay-hydrogel composite support-bath for bioprinting of complex structures. Sci. Rep..

[20] Zhao L (2019). A 3D printed hanging drop dripper for tumor spheroids analysis without recovery. Sci. Rep..

[21] Yumoto M (2020). Evaluation of the effects of cell-dispensing using an inkjet-based bioprinter on cell integrity by RNA-seq analysis. Sci. Rep..

[22] Ayan B (2020). Aspiration-assisted bioprinting for precise positioning of biologics. Sci. Adv..

